# Conjugated linoleic acid induces an atheroprotective macrophage MΦ2 phenotype and limits foam cell formation

**DOI:** 10.1186/s12950-015-0060-9

**Published:** 2015-02-19

**Authors:** Monica de Gaetano, Kawthar Alghamdi, Simone Marcone, Orina Belton

**Affiliations:** School of Biomedical and Biomolecular Science, UCD Conway Institute, University College Dublin, Dublin, Ireland; School of Medicine and Medical Science, UCD Conway Institute, University College Dublin, Dublin, Ireland

**Keywords:** Conjugated linoleic acid, Atherosclerosis, Macrophage differentiation, Foam cell formation, Scavenger receptors, Cholesterol efflux

## Abstract

**Background:**

Atherosclerosis, the underlying cause of heart attack and strokes, is a progresive dyslipidemic and inflammatory disease where monocyte-derived macrophage cells play a pivotal role. Although most of the mechanisms that contribute to the progression of atherosclerosis have been identified, there is limited information on those governing regression. Conjugated linoleic acid (CLA) is a group of isomers of linoleic acid that differ in the position and/or geometry of their double bonds. We have previously shown that a specific CLA blend (80:20 *cis*-9,*trans*-11:*trans*-10,*cis*-12-CLA) induces regression of pre-established atherosclerosis *in vivo, via* modulation of monocyte/macrophage function. However, the exact mechanisms through which CLA mediates this effect remain to be elucidated.

**Methods:**

Here, we address if CLA primes monocytes towards an anti-inflammatory MΦ2 macrophage and examine the effect of individual CLA isomers and the atheroprotective blend on monocyte-macrophage differentiation, cytokine generation, foam cell formation and cholesterol metabolism in human peripheral blood monocyte (HPBMC)-derived macrophages.

**Results:**

*cis*-9,*trans*-11-CLA and the atheroprotective 80:20 CLA blend regulates expression of pro-inflammatory mediators and modulates the inflammatory cytokine profile of macrophages and foam cells. In addition, *cis*-9,*trans*-11-CLA and CLA blend primes HPBMCs towards an anti-inflammatory MΦ2 phenotype, characterised by increased scavenger receptor (*CD36*) and efflux protein (*ABCA-1*) expression. Furthermore, this altered macrophage phenotype impacts on foam cell formation, inhibiting ox-LDL accumulation and promoting cholesterol efflux *via* both PPARγ and LXRα dependent pathways.

**Conclusion:**

The data increases the understanding of the pathways regulated by CLA in atheroprotection, namely, inhibiting the progressive acquisition of a pro-inflammatory macrophage phenotype.

**Electronic supplementary material:**

The online version of this article (doi:10.1186/s12950-015-0060-9) contains supplementary material, which is available to authorized users.

## Background

Biologically active macrophage cells play a pivotal role in atherosclerosis development from the early stages of monocyte to macrophage differentiation, intimal accumulation of macrophages and their conversion to foam cells *via* uptake of oxidised lipid, to the later stage of fibrous plaque development. In the context of atherosclerosis, the underlying cause of myocardial infarction and stroke, macrophages uniquely possess a dual functionality regulating and sustaining the chronic inflammatory response and regulating lipid accumulation and metabolism [1], two of the most well documented pathways associated with the pathogenesis of the disease.

At sites of atherosclerotic lesion development, LDL passively diffuses through the tight junction of the dysfunctional endothelium [2], where it becomes oxidised as a result of exposure to the oxidative stress of vascular cells thus giving rise to minimally oxidised-LDL which stimulates the overlying endothelial cells to express pro-inflammatory mediators which recruit and mediate the adhesion of leukocytes, primarily monocytes to the artery wall [3]. Monocytes adhere to the activated endothelial cells *via* specific interactions mediated by integrins [4] and following transendothelial migration, rapidly differentiate into macrophages in response to growth factors, such as macrophage colony-stimulating factor (M-CSF). M-CSF is required for the survival of both circulating monocytes and resident tissue macrophages [5] and potentiates a number of monocyte functions including phagocytic activity, microbial killing, tumor cell cytotoxicity, enhanced synthesis of inflammatory cytokines [6], and, of relevance to this study, differentiation of the monocyte cell into a mature macrophage. In atherosclerosis M-CSF mediates other macrophage-specific programmes, such as scavenger receptor (SR) and apo-E gene expression [7].

ox-LDL is taken up by the mature macrophage *via* scavenger receptor-mediated endocytosis, primarily CD36 and SR-A1, through endosomes. Endosomes-containing ox-LDL are trafficked to lysosomes where the cholesterol ester content of the ox-LDL is hydrolysed to fatty acids and free cholesterol, which is then trafficked out of the lysosome where it is re-esterified to cholesterol esters by acyl-coenzyme A:cholesterol acetyltransferase (ACAT). Cholesterol esters are stored in the cytosol or cleaved by the neutral cholesterol ester hydrolase to free cholesterol which is then effluxed from the macrophage *via* several transporters, including ATP-binding cassette (ABC) family (ABCA-1, ABCG-1) and SR-B1 to acceptor molecules, such as apo-A1 and HDL for subsequent metabolism in the liver [8,9]. This reverse cholesterol transport (RCT) pathway is regulated by the nuclear receptor Liver X Receptor (LXR).

During early stages of atherosclerosis, the concentrations of ox-LDL in the intima are sufficiently low for macrophage-mediated removal. However, as disease progresses and concentrations of ox-LDL increase, the balance between efflux and influx is altered, the RCT system is overwhelmed and free cholesterol is stored as cholesterol esters in the form of cytosolic lipid droplets. The accumulation of lipid droplets in the macrophage is indicative of foam cell formation and results in the fatty streak, the first clinical hallmark of atherosclerotic plaque [10].

Macrophages are heterogeneous cell populations, of which the pan macrophage marker is CD68, a glycoprotein present on the lysosomal membrane of the cell that adapts a response to environmental cytokines. Macrophages respond to stimuli from their micro-environment and, consequently, show high plasticity and heterogeneity [11]. The initial simplified classification of macrophage phenotypes is the discrimination between type-1 pro-inflammatory (MΦ1) and type-2 anti-inflammatory (MΦ2) macrophages on the basis of the cytokine environment created by two different classes of lymphocyte T helper (T_H_1 or T_H_2). Classically activated MΦ1 pro-atherogenic macrophages are primed by T_H_1 cytokines, such as IFN-γ and IL-1β, and function to increase and sustain the ongoing inflammatory response *via* production of pro-inflammatory mediators, such as TNF-α, IL-6, IL-1β and IL-12. Thus, continuous MΦ1 macrophage activity, contributes to tissue damage [1,12]. Alternatively activated MΦ2 anti-inflammatory macrophages are primed as a result of exposure to T_H_2 cytokines, such as IL-4 and IL-13 [13] and promote tissue repair and healing. Both MΦ1 and MΦ2 macrophages have been identified in human atherosclerotic plaque where MΦ2 macrophages are present at more stable locations [14]. More recently, it has been shown that the MΦ1 macrophage content of atherosclerotic plaques is associated with clinical incidence of ischemic stroke and increased inflammation [15]. Furthermore, it has been shown that there is an MΦ2 to MΦ1 switch during atherosclerotic plaque progression, suggesting that interventional tools which could revert the macrophage infiltrate towards the MΦ2 phenotype, may exert an atheroprotective action [16]. We have previously shown that, in a conjugated linoleic acid (CLA)-induced model of atherosclerosis *regression*, there is enrichment of MΦ2 genes in the aorta *in vivo* [17] which is in agreement with other studies which show that decreased lipid levels are associated with lower plaque lipid content and higher MΦ2 gene expression [18].

CLA is a family of naturally occurring geometric dienoic isomers of the ω6 essential fatty acid, linoleic acid (LA) [19]. CLA has a diverse range of benefits in health and diseases such as cancer [20,21], obesity [22,23], immune function [24] and atherosclerosis [25-28].

We have previously shown that dietary administration of a 1% CLA blend of the two most abundant isomers (80:20, *cis-*9,*trans-*11-CLA:*trans-*10,*cis-*12-CLA) induces regression of pre-established atherosclerosis in the apo-E^−/−^ mouse model, despite a continuing high cholesterol challenge [25], *via* modulation of monocyte/macrophage function [29,30].

Moreover, we have shown that CLA inhibits foam cell formation *in vitro*, *via* regulation of the nuclear receptor coactivator, peroxisome proliferator-activated receptor (*PPAR*)-γ coactivator (*PGC*)-1α [31]. Of relevance to this study, we have also shown that CLA increased macrophage polarization toward an anti-inflammatory MΦ2 phenotype *in vivo* [17] and that this is mediated *via PPARγ* dependent and independent mechanisms.

The effects of CLA on cholesterol homeostasis and foam cell formation have also been investigated in macrophage cell lines where *t*-10,*c*-12-CLA and *c*-9,*t*-11-CLA decreased foam cell formation [32] and increased expression of genes involved in RCT, such as *LXRα* and its target gene *ABCA-1*. In addition, levels of pro-inflammatory cytokine production (in particular TNF-α, IL-6 and IL-1β) were decreased following treatment with four different CLA isomers (*cis*-9,*trans*-11; *cis*-9,*cis*-11; *trans*-9,*trans*-11; *trans*-10,*cis*-12-CLA) in RAW-264.7 macrophages *via* a *PPARγ* dependent mechanism [33]. However, to date, studies with CLA on primary HPBMCs are limited.

The aim of this study is to investigate the effects of the atheroprotective CLA isomer *c*-9,*t*-11-CLA, the atheroprotective CLA blend (80:20 *c*-9,*t*-11:*t*-10,*c*-12-CLA) and the *t*-10,*c*-12-CLA isomer on the monocyte-macrophage-foam cell axis, specifically to identify changes in macrophage phenotype, inflammatory cytokine generation and cholesterol uptake and transport using HBPMC-derived macrophages, to ultimately further understand the mechanisms through which CLA mediates regression of pre-established atheroslerosis. Here, we provide evidence that CLA shifts HPBMC-derived macrophage differentiation to an anti-inflammatory phenotype, inducing expression of MΦ2 marker receptors and suppressing production of pro-inflammatory cytokines. Additionally, we show that CLA inhibits foam cell formation by reducing ox-LDL uptake and increasing cholesterol efflux. Our data describes a novel functional role for CLA in regulating macrophage phenotype and foam cell formation in the context of atherosclerosis regression.

## Methods

### Isolation of human peripheral blood monocytes

All experiments were conducted in conformity with institutional guidelines and in compliance with international laws. All volunteers gave written informed consent. Whole blood from healthy volunteers was drawn into heparin-coated vacutainers (BD, UK/Ireland). All volunteers were non-smoking, aged 25–30 years and free from medication for at least 10 days. Platelet-rich plasma (PRP) was isolated by centrifugation (190 × g for 15 min) and then diluted 1:3 with PBS before addition to Lymphoprep (Nycomed, Norway) and centrifuged at 450 × g for 30 min. Buffy-coats were recovered using a pasteur pipette, washed twice with PBS and resuspended in 10 ml serum-free medium (SFM) M-199 (Thermo Scientific), supplemented with L-glutamine (6.8 mM) and antibiotics (100 U/mL penicillin, 100 μg/mL streptomycin) and 10 ng/ml polymyxin-B-sulfate (Sigma-Aldrich, Dublin, Ireland). Monocytes were purified by plastic adherence in SFM for 2 hrs at 37°C, 5%CO_2_.

### HPBMC-derived macrophage differentiation and cell treatment

Prior to *in vitro* experiments, macrophage differentiation of freshly isolated HBPMCs was stimulated with 100 ng/ml M-CSF (Gibco-BRL Life Technologies Ltd, England, UK), in 10% human serum (HS) (Sigma-Aldrich, Dublin, Ireland). After four days of culture at 37°C, 5%CO_2_, media was changed and a further 100 ng/ml M-CSF was added in 10% HS (Additional file 1).

For experiments on differentiating macrophages, cells were washed twice with warm PBS and treated for 48 hrs at 37°C, 5%CO_2_, in 1% HS, with 10 μM of *cis-9,trans-11-*CLA, *trans-10,cis-12-*CLA , CLA blend (80:20 *c-9,t-11*:*t-10,c-12*); linoleic acid (LA) and oleic acid (OA) (all Cayman Chemicals, MI, USA); 5 μM PPARγ agonist, troglitazone (TROG) or dimethyl sulfoxide (DMSO) (vehicle control) (both Sigma-Aldrich, Dublin, Ireland) at day 4 of culture. At day 6 cells were fixed for immunocytochemistry or lysed for mRNA analysis.

For experiments on mature macrophages and foam cell formation, media was changed and further 100 ng/ml M-CSF were added in 10% HS after four days of culture at 37°C, 5%CO_2_. Cells were treated with CLA and controls as above at day 8 and fixed at day 10. Where relevant, cells were also treated with 1 μM LXRα agonist T0901317 (T1317) or 10 μM 25-hydroxycholesterol (25-OH) (both from Sigma-Aldrich, Dublin, Ireland).

### HPBMC-derived foam cell formation

After ten days of culture, as above described, mature macrophage were washed twice with warm PBS and incubated for further 4 hrs with 50 μg/ml of human ox-LDL or human fluorescently labelled Dil-ox-LDL (both Intracel MD, USA).

Following CLA and control treatments lipid loading, wells were washed three times with fresh medium and fluorescent Dil emission was measured in a Spectramax M2 (Molecular devices, CA, USA) plate fluorescence reader with 550/568 nm excitation/emission wavelength. The levels of Dil-ox-LDL were adjusted per cell number by measuring the intensity of DAPI fluorescence, with a second reading at 360/460 nm. Ox-LDL uptake measurements were repeated in triplicate in three independent experiments and the mean value was expressed as a percentage of vehicle control.

### THP-1 cell culture and cell treatments

Human THP-1 monocytes (ATCC) were cultured in RPMI 1640 medium supplemented with fetal bovine serum (10%), penicillin (100 U/ml), streptomycin (100 ug/ml) and L-glutamine (2 mM) (Gibco BRL, UK). THP-1 monocytes (1 × 10^6^) were differentiated to macrophages using 100 nmol/L phorbol 12-myristate 13-acetate (PMA) (Sigma Aldrich, Dublin, Ireland) for 72 hrs.

THP-1 macrophage were treated for 18 hrs with DMSO, 25 μM of *c*-9,*t*-11-CLA and CLA blend, TROG (10 μM), T1317 (1 uM), alone or pre-incubated for 2 hrs with 10 μM of the PPARγ inhibitor, GW9662 (GW) (Cayman Chemicals, MI, USA), or 1 μM of the LXRα inhibitor, GSK2033, (GSK) (Axon).

### RNA isolation and gene expression analysis

For gene expression experiments, HBPMC- or THP-1-derived macrophages and HPBMC-derived foam cells were washed twice with ice cold PBS, prior to addition of 200 μl of RLT buffer (Qiagen, UK). Total RNA was isolated from cell lysates using the RNeasy kit (Qiagen, UK) as per manufacturers’ instructions. Reverse transcription was carried out on 1 μg of total RNA using Superscript^TM^ III Reverse Transcriptase (Invitrogen) according to the manufacturers’ instructions. Relative gene expression quantification by real-time PCR (RT-PCR) was performed on an ABI Prism 7900HT Sequence Detection System (Applied Biosystems Inc., UK). *MR* and *SRA-1* expression were examined using specific Taqman assays (Applied Biosystems Inc., UK), whilst, *ABCA-1*, *CD36*, *CD14*, *CD68* and *CD163* were measured using specific Syber green assays (Applied Biosystems Inc., UK) (Additional file 2). Ct values were normalised to 18s ribosomal RNA.

### Immunocytochemistry

For visualisation of HPBMC-derived macrophages and foam cells, 5x10^5^ cells were seeded onto glass coverslips placed in 12-well plate and treated, as above, over 6 days of culture. Mature macrophages or ox-LDL loaded-foam cells were then fixed in 3% formaldehyde (Sigma-Aldrich, Dublin, Ireland), permeabilized with Triton-X-100 (Sigma-Aldrich, Dublin, Ireland). Non-specific binding was prevented by blocking with 5% BSA (Sigma-Aldrich, Dublin, Ireland). Target proteins were then labelled using 1:100 of anti-human goat polyclonal CD68 and goat polyclonal MR (Sigma-Aldrich) and fluorescently labelled-secondary antibodies (1:200 of Alexa-Fluor 488, 568 or 647) (Invitrogen, Carlsbad, California), followed by Alexa Fluor 568-phalloidin staining (Invitrogen, Carlsbad, California), for F-actin, and DAPI (Sigma-Aldrich, Dublin, Ireland). For foam cell visualization, only DAPI staining was required as fluorescently labelled ox-LDL was used (Dil emission at 568nm). Cells were imaged using a Zeiss AxioImager M1 fluorescent microscope and the images were captured using an Olympus digital camera (Optronics, Goleta, CA).

### Intracellular cholesterol measurement

Intracellular cholesterol levels were measured using the Amplex® Red Cholesterol Assay kit (Molecular Probes), based on an enzyme-coupled reaction that detects both free and esterified cholesterol.

After 10 days of culture and treatment with CLA isomers and controls as described above, HPBMC-derived macrophages and foam cells were lysed using T-PER. Firstly, a cholesterol standard curve was prepared, diluting the cholesterol reference standard. The Amplex Red reagent, containing the substrate HRP and both cholesterol oxidase/esterase, enzymes was prepared according to the manufacturers’ guidelines. 50 μl Amplex Red reagent was added to each sample and the samples were then incubated for 30 mins at 37°C. Fluorescence was measured using a microplate reader, using excitation in the range of 530–560 nm and emission detection at ~590 nm. Each point was corrected for background fluorescence by subtracting the values derived from the no-cholesterol control.

### ELISA for cytokine quantification

Following 10 days of differentiation firstly to macrophages, and subsequenty to foam cells following exposure to ox-LDL, in the presence of CLA isomers or appropriate controls, as described above, HBPMC-derived macrophage/foam cell supernatants were collected and cleared by centrifugation (10,000 × g for 10 min at 4°C). The concentration of IL-10, IL-6, IL-1β, INF-γ, TNF-α and IL12-p70 in conditioned media was determined by enzyme immunoassay (EIA) using commercially available human 96 well-plate multiplex kit for tissue culture samples (MSD, Gaithersburg, MD, USA) according to the manufacturers’ guidelines.

### Statistical analysis

Results are expressed as mean ± SEM or fold change relative to vehicle control. Experimental points were performed in triplicate with a minimum of three independent experiments (n = 3). Statistical comparisons between controls versus treated groups were made by Student’s unpaired t-test, assuming unequal variance with a two-tailed distribution. A value of p < 0.05 or greater were considered significant.

## Results

### CLA primes human monocytes towards an MΦ2 phenotype

**Figure 1:**
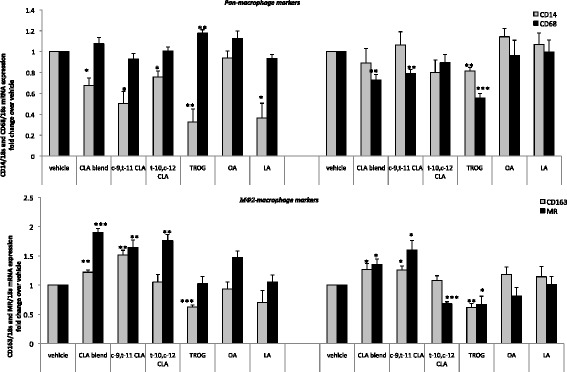
**CLA primes monocytes to an anti-inflammatory MΦ2 macrophage phenotype.** RT-PCR analysis of **(a)** *CD14* and *CD68* and **(b)** *CD163* and *MR* mRNA expression in or M-CSF stimulated (100 ng/ml) differentiating HPBMCs following treatment with *c*-9,*t*-11-CLA, *t*-10,*c*-12-CLA, CLA blend (80:20 *c*-9,*t*-11:*t*-10,*c*-12), OA, LA or TROG. In unstimulated conditions, both CLA isomers and their blend decrease *CD14* expression and increase expression of both *CD163* and *MR*, without affecting *CD68* expression. Following M-CSF stimulation, *c*-9,*t*-11-CLA and CLA blend decrease the mature macrophage marker *CD68* and increase expression of both *CD163* and *MR* (MΦ2 markers). Data are mean +/− SEM of three independent experiments. Data is expressed as fold change in gene expression relative to DMSO control, where *p < 0.05; ** p < 0.01 and ***p < 0.001 vs DMSO.

To elucidate if the atheroprotective CLA blend (80:20 *c*-9,*t*-11:*t*-10,*c*-12-CLA) impacts on monocyte-macrophage differentiation, we analysed the mRNA expression of the pan-monocyte and pan-macrophage markers, *CD14* and *CD68*, respectively (Figure 1a), and of the MΦ2-type macrophage markers mannose receptor (MR) and *CD163* (Figure 1b) in unstimulated or M-CSF-stimulated HPBMCs. Unstimulated monocyte cells were maintained in culture for 6 days, as a model of early macrophage differentiation, and M-CSF treated HPBMCs were used as a model of mature differentiated macrophages. Following 6 days of culture, unstimulated and M-CSF-stimulated macrophages were treated with 10 μM *cis-*9*,trans-*11*-*CLA (*c*-9,*t*-11-CLA), *trans-10,cis-12-*CLA (*t*-10,*c*-12-CLA), CLA blend (80:20 *c-9,t-11*:*t-10,c-12*), linoleic acid (LA), oleic acid or 5 μM of the PPARγ agonist, troglitazone (TROG) and analysed for expression of macrophage markers by RT-PCR and fluorescent microscopy.

In unstimulated conditions, both the individual CLA isomers and their 80:20 blend significantly decreased *CD14* mRNA expression (*c*-9,*t*-11-CLA by 2 fold, p < 0.05; *t*-10,*c*-12-CLA by 1.3 fold, p < 0.05 and 80:20 CLA blend by 1.4 fold, p < 0.05) *via* a PPARγ dependent mechanism, where the PPARγ agonist TROG, also decreased *CD14* expression (by 3.3 fold, p < 0.01). Although the lipid control OA had no effect, the parent compound, LA, also decreased *CD14* expression, suggesting a general class effect of linoleic acids on unstimulated monocytes in early differentiation. The effect of CLA in repressing *CD14* expression in early differentiation, was not maintained upon M-CSF treatment. This is expected, as *CD14* is a monocyte marker which is not highly expressed in mature differentiated macrophages. Interestingly, the PPARγ agonist maintained a decrease in *CD14* expression (by 1.3 fold, p < 0.01) suggesting that activation of PPARγ may inhibit the process of monocyte to macrophage differentiation even in the presence of M-CSF. Although the pan-macrophage marker *CD68* was not regulated by any lipid treament in unstimulated macrophages, following stimulation with M-CSF, both *c*-9,*t*-11-CLA and CLA blend decreased *CD68* expression compared to DMSO control (by 1.3 fold, p < 0.01 and by 1.4 fold, p < 0.01, respectively) *via* a PPARγ dependent mechanism, where TROG also decreased *CD68* expression (by 1.8 fold, p < 0.001). Conversely, *t*-10,*c*-12-CLA, OA and LA had no effect on CD68 expression (Figure 1a). This suggests a specificity of the atheroprotective CLA isomer (*c*-9,*t*-11) and the CLA blend in alteration of macrophage phenotype, following monocyte differentiation. This is in keeping with previous studies which show that the *c*-9,*t*-11 isomer, but not the *t*-10,*c*-12 isomer, is atheroprotective [34-36].

In these experiments, CLA was added after four days of culture, providing a model to investigate how CLA primes monocytes during their differentiation to macrophages. Therefore, this data suggests that treatment with the atheroprotective CLA blend and the *c*-9,*t*-11 isomer may prime monocytes towards an MΦ2 macrophage phenotype. This was confirmed by analysis of the expression of the MΦ2-type marker, *CD163* receptor, which also indirectly contributes to an anti-inflammatory response [37]. In both the presence and absence of M-CSF, *c*-9,*t*-11-CLA and CLA blend increased *CD163* expression (in unstimulated cells by 1.5 fold, p < 0.01 and by 1.2 fold, p < 0.01, respectively; in M-CSF stimulated cells by 1.3 fold, p < 0.05 for both treatments). Interestingly, this effect was independent of PPARγ, as TROG decreased *CD163* expression in both the absence and presence of M-CSF. Under both conditions, *t*-10,*c*-12-CLA, OA and LA had no effect on target gene expression, again confirming specificity for the atheroprotective isomers in altering macrophage phenotype (Figure 1b).

MR mediates the endocytosis of glycoproteins by macrophages [38,39] and is a well characterised MΦ2-type phenotype marker [8,14,40].

mRNA analysis of *MR* showed that, in both unstimulated and M-CSF-stimulated conditions**,***c*-9,*t*-11-CLA and CLA blend increased *MR* expression. In the absence of M-CSF, *MR* expression was increased by *c*-9,*t*-11-CLA and CLA blend (by 1.6 fold, p < 0.01 and 1.9 fold, p < 0.001, respectively) and this effect was maintained upon stimulation with M-CSF (*c*-9,*t*-11-CLA by 1.6 fold, p < 0.05 and the CLA blend by 1.3 fold, p < 0.05) (Figure 1b). In contrast, although *t*-10,*c*-12 increased *MR* expression by 1.8 fold (p < 0.01), in unstimulated conditions, this effect was not observed upon stimulation with M-CSF. Furthermore, neither OA nor LA had any effect on *MR* expression.

**Figure 2:**
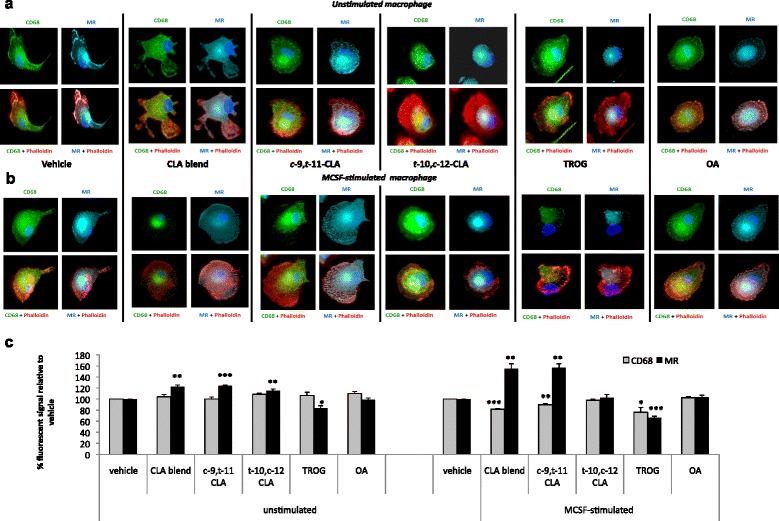
**CLA inhibits CD68 and increases MR expression in HPBMC-derived macrophages. (a)** Freshly isolated HBPMCs were cultured for four days in the presence or absence of M-CSF and treated with *c*-9,*t*-11-CLA, *t*-10,*c*-12-CLA, CLA blend (80:20 *c-*9,*t-*11:*t-*10,*c-*12), OA, LA or TROG for a further 48 hrs. At day 6, macrophage were fixed and stained. Immunofluorescence analysis of unstimulated macrophage shows that both CLA isomers and their blend increase MR expression but do not affect CD68 expression. **(b)** Differentiation of HBPMCs in the presence of 100 ng/ml M-CSF showed that *c*-9,*t*-11-CLA and CLA blend inhibits CD68 expression and increases MR expression. Epifluorescent microscope images (63× magnification) are representative of three independent experiments. Blue indicates DAPI (nuclei), green indicates CD68 (macrophage marker), cyan indicates MR and red indicates phalloidin (cytoplasmic) staining. **(c)** Spectrophotometry quantification of CD68 and MR fluorescent signal confirms the regulation of CD68 and MR by *c*-9,*t*-11-CLA and the atheroprotective blend. Statistical analysis of three independent experiments is expressed mean % as fluorescence ± SEM *vs* vehicle where *p < 0.05; **p < 0.01 and ***p < 0.001.

To validate the hypothesis that transcriptional changes may influence protein expression, we also performed immunocytochemistry of the pan-macrophage marker CD68 and of the MΦ2-type receptor MR, in HPBMCs either unstimulated (Figure 2a) or under M-CSF-stimulated conditions (Figure 2b). Immunofluorescence microscopy and subsequent spectrophotometric quantification of the fluorescent signals (Figure 2c) showed that, in unstimulated conditions, CLA had no effect on CD68 expression but upon M-CSF stimulation, both the *c*-9,*t*-11 isomer and CLA blend decreased CD68 (by 10 ± 1%, p < 0.01 and 18 ± 1%, p < 0.001, respectively). Importantly, in mature macrophages following M-CSF stimulation, *c*-9,*t*-11-CLA and CLA blend increased MR expression (by 57 ± 7% p < 0.01 and 55 ± 9%, p < 0.01, respectively). Finally, *t*-10,*c*-12-CLA, OA and linoleic acid had no effect on *CD68* or MR expression under either condition. This confirms that the atheroprotective CLA isomer and blend primes monocytes to an MΦ2 phenotype during differentiation, thus altering the macrophage phenotype. Negative controls for immunofluorescence experiments are shown in “Additional Information” (Additional file 3).

### CLA reduces foam cell formation *via* a PPARγ/LXRα-dependent regulation of cholesterol metabolism

LXRα, LXRβ and PPARγ are ligand-activated nuclear receptors controlling cholesterol distribution and efflux, the inflammatory response and, in the case of PPARγ, macrophage polarization state. As described above, the effect of CLA on priming monocytes towards an MΦ2 phenotype is mediated in part *via* a PPARγ dependent mechanism. To understand the functional consequences of CLA in priming monocytes to alternatively activated macrophages, in the next set of experiments, we investigated the effect of CLA on foam cell formation in HPBMC-derived macrophages. Freshly isolated HPBMCs were differentiated towards a macrophage phenotype using 100 ng/ml M-CSF. After 8 days of culture cells were treated for 48 hrs with CLA or controls. To investigate the putative role of PPARγ and LXRα receptor in mediating the effect of CLA, the synthetic PPARγ agonist, TROG (10 μM) and LXRα agonist, T0901317 (also known as T1317) (1 μM) were used as controls. Following treatment, mature macrophages were loaded with 50 μg/ml Dil-ox-LDL for 4 hrs to induce foam cell formation. Similar to what we previously observed in RAW macrophage cells [31], immunofluorescence microscopy and its relative quantification clearly showed that both *c*-9,*t*-11-CLA and *t*-10,*c*-12-CLA isomers, as well as their 80:20 blend, decreased ox-LDL uptake (by 23 ± 3%, p < 0.01; 15 ± 4%, p < 0.05 and 16 ± 4%, p < 0.05, respectively), whereas LA control had no effect. Interestingly, both the PPARγ and LXRα agonists also inhibited foam cell formation (by 38 ± 2%,p < 0.001 and 36 ± 11%, p < 0.05, respectively), suggesting a dual PPARγ/LXRα-dependent mechanism, through which CLA inhibits foam cell formation (Additional file 4).

**Figure 3:**
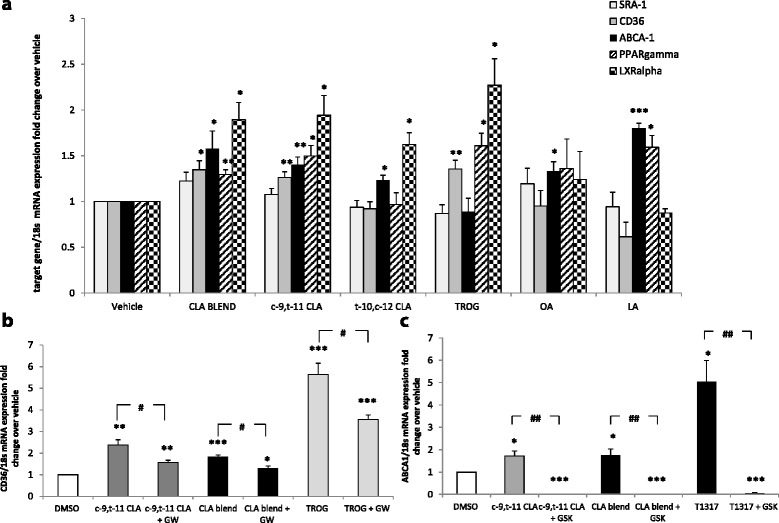
**CLA increased** ***CD36*** **and** ***ABCA-1*** **expression** ***via*** **a PPARγ/LXRα mechanism. (a)** RT-PCR analysis of *SRA-1*, *CD36*, *ABCA-1*, *PPARγ* and *LXRα* in HPBMC-derived macrophages pre-treated with *c*-9,*t*-11; *t*-10,*c*-12; CLA blend; OA; LA; TROG or T1317 and stimulated with ox-LDL for 4 hours to induce foam cell formation. CLA blend and *c*-9,*t*-11 increase *CD36* and *ABCA-1*. Although *t*-10,*c*-12 has no effect on SR expression, it incresases *ABCA-1*, which is a generalized effect of linoleic acids. The same effect was observed with the parent compound LA. RT-PCR analysis of **(b)** *CD36* and **(c)** *ABCA-1* mRNA expression in PMA-induced macrophages treated with *c*-9,*t*-11, CLA blend and TROG alone or in combination with the PPARγ and LXRα antagonists (GW9662 and GSK2033, respectively). Pre-treatment with the antagonists attenuates or abolished the CLA-induced upregulation of both *CD36* and *ABCA-1*, respectively. Statistical analysis of three independent experiments is expressed as fold change expression relative to DMSO control where *p < 0.05; **p < 0.01 and ***p < 0.001 or relative to the combination of the antagonist and the antagonist *vs* the agonist alone, where ^#^p < 0.05 or ^##^p < 0.01.

To elucidate the mechanism through which CLA, by inducing an MΦ2 phenotype, inhibits foam cell formation, we next examined expression of the scavenger receptors *SR-A1* and *CD36* in human macrophage-derived foam cells (Figure 3a). Although CLA had no effect on *SR-A1* expression, both *c*-9,*t*-11-CLA and CLA blend increased *CD36* expression in the presence of ox-LDL (by 1.3 fold, p < 0.01 for both), whereas, neither *t*-10,*c*-12-CLA isomer nor either of the two fatty acid controls modulated *CD36* expression.

Furthermore, both CLA isomers and their 80:20 blend increased *ABCA-1* mRNA expression (by 1.4 fold, p < 0.01; 1.2 fold, p < 0.05 and 1.6 fold, p < 0.05, respectively). However, the fatty acid controls also increased *ABCA-1* expression, suggesting that the induction of efflux proteins is a generalised fatty acid effect, whereas, the regulation of *CD36* is unique to the atheroprotective CLA isomers and blend. To identify the mechanisms involved in increased *CD36* and *ABCA-1* levels, expression of the nuclear receptors, which regulate cholesterol transport and macrophage polarization, were investigated in both the presence and absence of CLA. In ox-LDL treated macrophages, *c*-9,*t*-11-CLA and the 80:20 CLA blend increased *PPARγ* expression (by 1.5 fold, p < 0.05 and by 1.3 fold, p < 0.05 respectively). As expected, the receptor agonist TROG also increased its expression by 1.6 fold (p < 0.05). Furthermore, CLA isomers and their blend, as well as the *PPARγ* agonist, induced a marked increase in the expression of the nuclear receptor *LXRα* (*c*-9,*t*-11-CLA and CLA blend by 1.9 fold, p < 0.05; *t*-10,*c*-12-CLA by 1.6 fold, p < 0.05 and TROG by 2.3 fold, p < 0.05). This is a potent and specific effect of CLA, as neither fatty acid control affected expression. As positive controls, the effects of two LXRα agonists, T1317 and 25-OH, were verified on the nuclear receptor LXRα and on its target gene *ABCA-1* in HBPMCs-derived macrophages. As expected, both LXRα agonists increased *LXRα* expression (Additional file 5). Taken together, this data strongly suggests that the atheroprotective *c*-9,*t-*11-CLA and CLA blend-mediated regulation of *CD36* and ABCA-1 expression is *via* PPARγ and LXRα dependent mechanisms.

To confirm our hypothesis, we extended our studies to examine the effect of the atheroprotective CLA isomer and the blend on the expression of *PPARγ* and *LXRα* target genes *CD36* and *ABCA-1*, in the presence and absence of PPARγ or LXRα antagonists, in a distinct model of macrophage differentiation, namely PMA-induced THP-1 macrophage formation (Figure 3b and c). As confirmation of a CLA PPARγ − dependent mechanism, pre-incubation with the PPARγ antagonist, GW9662, attenuated the effect of both *c*-9,*t*-11-CLA and CLA blend (by 0.3 fold, p < 0.05, for both) on *CD36* expression, similar to that observed when the PPARγ agonist, TROG and the antagonist GW9662 were combined (by 0.4 fold, p < 0.05). This provides convincing evidence that regulation of *CD36* expression by *c*-9,*t*-11 and CLA blend is PPARγ dependent. Importantly, the CLA-induced increased expression of ABCA-1 was completely abolished by pre-incubation with the LXRα antagonist GSK2033 (by 1 fold, p < 0.01, for both), similar to that observed when the LXRα agonist, T1317, and the antagonist GSK2033 were combined (by 1 fold, p < 0.01). This provides convincing data that CLA mediated regulation of *ABCA-1* expression is LXRα dependent. These experiment confirm the specificity of CLA in modulation of macrophage function *via* both PPARγ and LXR-dependent mechanisms.

### CLA reduces intracellular cholesterol in foam cells

As CLA alters the macrophage to an MΦ2 MR^+ve^ phenotype, we hypothesized that a potential functional effect of CLA on MΦ2 macrophages in response to ox-LDL and the inhibition of foam cell formation, is the reduction of intracellular cholesterol by promoting efficient cholesterol metabolism.

**Figure 4:**
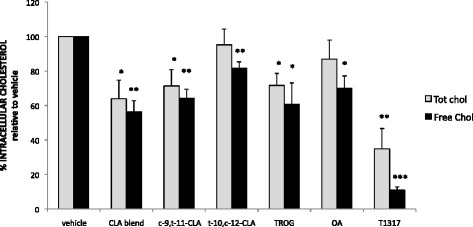
**CLA reduces intracellular cholesterol content of human macrophage derived foam cells.** HBPMCs-derived M-CSF stimulated macrophages, following treatment with *c*-9,*t*-11-CLA, *t*-10,*c*-12-CLA, CLA blend (80:20 *c*-9,*t*-11:*t*-10,*c*-12), OA, LA or TROG were ox-LDL loaded for 4 hours and cholesterol content measured by enzymatic assay. CLA blend and *c*-9,*t*-11-CLA significantly decrease the levels of both total cholesterol (TC) and free cholesterol (FC), whilst, *t*-10,*c*-12-CLA and OA decrease the FC fraction. The effect of CLA on cholesterol trafficking is regulated by a dual mechanism involving PPARγ/LXRα-dependent. Data are expressed as % of TC or FC content over vehicle control, where *p < 0.05, **p < 0.01 and are the mean of three independent experiments.

To address our hypothesis, we investigated the effect of CLA on intracellular cholesterol trafficking. To this end, HBPMCs-derived MCSF-induced macrophages were analysed for intracellular cholesterol content, following CLA treatment and subsequent ox-LDL loading, using the Amplex red cholesterol assay. Subsequently, we measured the fluorescent signals generated by the activity of both cholesterol oxidase and esterase, calculating the content of both free cholesterol (FC) and total cholesterol (TC). *c*-9,*t*-11-CLA significantly decreased the levels of TC by 29 ± 9% (p < 0.05) and of FC by 36 ± 5% (p < 0.01) and CLA blend reduced TC by 36 ± 11% (p < 0.05) and FC by 44 ± 7% (p < 0.01). Importantly, neither the *t*-10,*c*-12-CLA isomer nor OA had any significant effect on TC although they both reduced FC. The PPARγ agonist TROG also reduced TC by 28 ± 7% (p < 0.05) and FC by 39 ± 12% (p < 0.05) and the LXRα agonist T1317 reduced TC by 65 ± 12% (p < 0.01) and FC by 89 ± 2% (p < 0.001). This data confirms that the overall effect of the *c*-9,*t*-11-CLA isomer and the CLA blend on cholesterol trafficking is regulated by a dual mechanism: a PPARγ-dependent pathway, controlling cholesterol influx (as previously confirmed by the regulation of *CD36*) and an LXRα-dependent mechanism, directly influencing the cholesterol efflux (through upregulation of the efflux protein *ABCA-1*, as previously shown) (Figure 4).

### CLA inhibits production of pro-inflammatory cytokines in human macrophages and foam cells

It is now widely accepted that the cytokine microenvironment plays a fundamental role in the priming and switching of MΦ1/MΦ2 macrophage subtypes [41].

**Figure 5:**
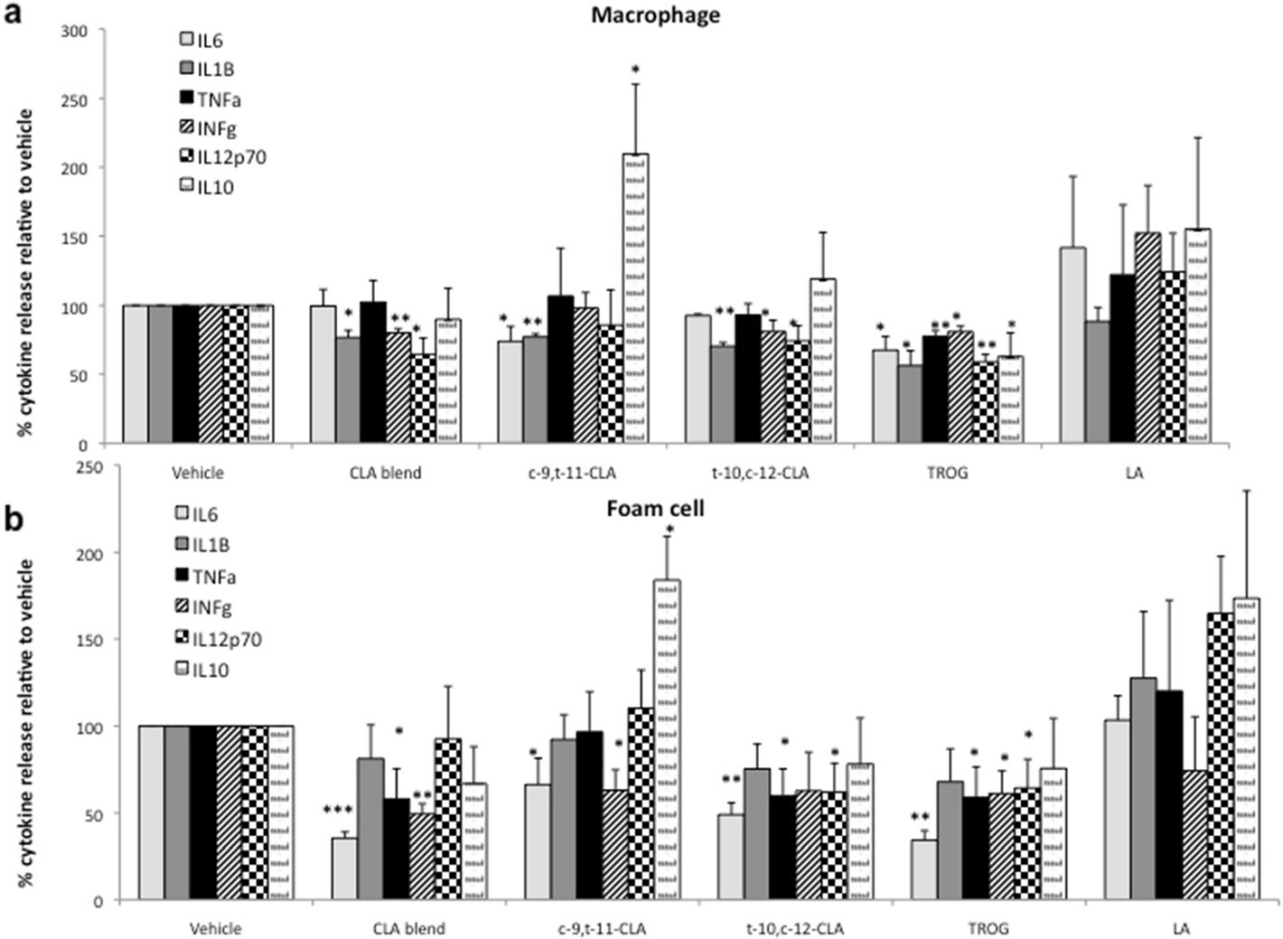
**CLA inhibits production of pro-inflammatory citokine secretion in human macrophages and foam cells.** Measurement of cytokine released was quantified in HPBMC-derived **(a)** macrophages and **(b)** foam cells following treatment with *c*-9,*t*-11; *t*-10,*c*-12; CLA blend; OA; LA or TROG using a multiplex ELISA. Both individual CLA isomers and the CLA bend reduced pro-inflammatory cytokine generation *via* a PPARγ dependent mechanisms. Uniquely, *c*-9,*t*-11-CLA increased macrophage and foam cell generation of IL10, *via* a PPARγ independent mechanism. Data are expressed as % cytokine release over vehicle control and are the mean of three independent experiments where *p < 0.05, **p < 0.01, ***p < 0.001.

Using multiplex ELISA, we showed that pro-inflammatory cytokine generation was reduced in mature macrophages by CLA, in a similar manner to that of the PPARγ agonist (TROG) (Figure 5a). *c*-9,*t*-11-CLA significantly inhibited IL-6 and IL-1β generation (by 26 ± 10%,p < 0.01 and by 23 ± 2%, p < 0.05, respectively) and the CLA blend inhibited IL-1β, INF-γ and IL-12p70 (by 23 ± 5%,p < 0.05; by 20 ± 3%, p < 0.01 and by 36 ± 12%, p < 0.05, respectively). Interestingly, *t*-10,*c*-12-CLA also reduced IL-1β, INF-γ and IL-12p70 (by 30 ± 3%,p < 0.01; by 19 ± 8%, p < 0.05 and by 26 ± 11%, p < 0.05, respectively). Coincident with its role in macrophage polarisation, the PPARγ agonist inhibited all pro-inflammatory cytokines analysed. Importantly, *c*-9,*t*-11-CLA also increased anti-inflammatory cytokine IL-10 generation in macrophages (by 2.1 fold, p < 0.05), suggesting a specificity of this isomer to profoundly alter the cytokine profile of the marophage cells. Furthermore, we showed that the altered cytokine profile was maintained following stimulation of foam cell formation where pro-inflammatory cytokines generation was reduced by CLA, in a similar manner to that of the PPARγ agonist (TROG) (Figure 5b). In particular, *c*-9,*t*-11-CLA significantly inibited IL-6 and INF-γ generation (by 34 ± 15%, p < 0.05 and by 37 ± 12%, p < 0.05, respectively) and the CLA blend inibited IL-6, TNF-α and INF-γ (by 65 ± 4%,p < 0.001; by 42 ± 17%, p < 0.05; by 49 ± 6%, p < 0.01, respectively). *t*-10,*c*-12-CLA also reduced IL-6, TNF-α and IL-12p70 (by 51 ± 7%,p < 0.01; by 40 ± 16%, p < 0.05 and by 38 ± 17%, p < 0.05, respectively) and TROG reduced generation of IL-6, TNF-α, INF-γ and IL12p70.

Again, *c*-9,*t*-11-CLA increased IL-10 generation in foam cells by 1.8 fold (p < 0.05). Finally, fatty acid control LA had no effect on any of the cytokines analyzed. In summary, in both macrophage and foam cell, generation of pro-inflammatory cytokines was decreased by CLA in a similar manner to that of the PPARγ agonist (TROG) and *c*-9,*t*-11 isomer increased the generation of anti-inflammatory IL-10.

## Discussion

Over the last decade, in parallel with the discovery of several anti-atherogenic properties of CLA [27,28], several studies have focused on identifying the cellular mechanism through which CLA mediates its effect with several lines of evidence, including our previous studies, suggesting that it is *via* the monocyte/macrophage cell [4,17,25,26,29-31]. To date, the effect of CLA on the modulation of macrophage/foam cell phenotype and function have primarily been conducted in cell lines, in particular the murine RAW-264.7 [35,36] and the human THP-1 cells [29], as well as in murine [27] and rabbit models of atherosclerosis [21,28,42]. Not surprisingly, the use of *in vivo* models, *ex vivo* cell culture systems and/or *in vitro* immortalised cell lines, as well as different isomeric blends of CLA, have resulted in conflicting data in relation to the effect of CLA on macrophage function.

In this study, we used a translationally relevant cell model, namely human peripheral blood monocytes, to elucidate the effect of CLA on macrophage plasticity, foam cell formation, intracellular cholesterol metabolism and cytokine generation. Importantly, we examined the effect of the 80:20 blend, previously shown by us to induce regression and inhibit foam cell formation [25,31], as well as the atheroprotective isomer *c*-9,*t*-11 which inhibits monocyte adhesion/migration [4,29]. We also examined the *t*-10,*c*-12 isomer as a control, which has previously been shown to have no effect and, in some studies, in fact, increases atherosclerosis [43]. Although CLA is a known ligand for the nuclear receptor PPARγ [44,45] and is thus a potential pathway through which CLA mediated its atheroprotective effects [33], we have recently shown that the effect of CLA on monocyte function is mediated *via* both PPARγ dependent and independent mechanisms [4] and, in this study, we identify that CLA mediates its effects on the macrophage/foam cell *via* an additional LXRα dependent mechanism.

Macrophage differentiation is diversely regulated by several growth factors [46]. A major aim of this study was to examine if CLA primes human monocytes to adopt an MΦ2 phenotype. To address this, we used M-CSF, a known inducer of macrophage polarization [47] and the effect of CLA isomers and the atheroprotective blend on the mRNA expression of macrophage markers was analysed, in the presence or absence of M-CSF stimulation. We show that, during early stages of monocyte-macrophage differentiation, modeled *in vitro* by the absence of M-CSF, both CLA isomers and the 80:20 blend decreases *CD14*, a receptor specific to the “monocytic phase” of the cell [48], whilst, as differentiation progresses, the atheroprotective CLA isomer decrease *CD68* expression, a receptor which characterizes later stages of macrophage cell development [40]. Of importance is the observation that CLA also upregulates the specific MΦ2-type markers *CD163* and *MR* [14], priming a switch towards an anti-inflammatory macrophage phenotype. Our data suggest a potential PPARγ-dependent mechanism of CLA in the regulation of the aforementioned receptors. Indeed, this is supported by Bouhlel et al., who showed that PPARγ activation primes human monocytes into alternative macrophage, characterized by high levels of *MR*, positively related to highly expressed PPARγ [14].

In the absence of differentiating stimulus, in early monocyte-macrophage differentiation, both *c*-9,*t*-11-CLA and the atheroprotective CLA blend promoted a CD68^high^/MR^high^ phenotype, whilst, when M-CSF-triggered, switched to a CD68^med^/MR^high^ and a CD68^low^/MR^high^ phenotype, thereby also regulating CD68 protein expression. Moreover, stimulation with M-CSF, amplifies the effect of *c*-9,*t*-11-CLA on the mannose receptor, which although expressed in resting conditions, albeit at very low levels, is significantly increased following treatment. Importantly, this data identifies a putative atheroprotective role for CLA, specifically *c*-9,*t*-11 isomer, in priming monocytes towards MΦ2 macrophage phenotype.

To fully understand the functional consequences of altered macrophage phenotype on foam cell formation, HPBMC-derived macrophages were pre-treated with CLA isomers prior to the addition of the pro-atherogenic ox-LDL. *c*-9,*t*-11-CLA and CLA blend decreased foam cell formation, and *t*-10,*c*-12-CLA isomer induced a similar, although less pronounced effect. Interestingly, both agonists of PPARγ and LXRα inhibited foam cell formation, suggesting a possible dual PPARγ/LXRα-dependent mechanism, through which CLA prevents foam cell formation. This is in keeping with our recent study on RAW macrophage cells, which shows that CLA mediates its effect on foam cell formation *via* regulation of *PGC-1α*, the transcriptional activator of both PPARγ, LXR and other nuclear receptors [31].

The canonical route for ox-LDL to enter the cell is *via* scavenger receptor-mediated uptake and inhibition of *CD36* and *SRA-1* scavenger receptor expression has been shown to limit foam cell formation [49]. However, a second potential mechanism for the inhibition of foam cell formation is *via* the RCT system which removes cholesterol to HDL for metabolism in the liver. Primarily, the ABC transporters, including ABCA-1, regulates RCT. Previous studies have shown that LXRα regulates cholesterol trafficking *via* modulation of ABC efflux proteins [8]. Thus, to better understand the mechanism through which CLA inhibits foam cell formation, both uptake and efflux of ox-LDL was examined, initially by analysing the expression of *SR-A1*, *CD36* [49,50] and *ABCA-1* [9,51] following ox-LDL loading of mature HPBMC-derived macrophage. Furthermore, using the PMA-induced THP-1 macrophage cell line model, regulation of the aforementioned two nuclear receptors’ target genes *CD36* and *ABCA-1* expression was also verified in the presence of CLA isomers alone or in combination with PPARγ and LXRα antagonists, which, respectively, attenuates or abolished the CLA-induced upregulation of those genes. This data confirms that ox-LDL influx and efflux is controlled by a dual PPARγ/LXRα-dependent mechanism.

Our data shows that CLA inhibits foam cell formation, in the presence of an exogenous source of oxidized lipoproteins, by increasing expression of the PPARγ target *CD36*, thus promoting the uptake of the relatively abundant presence of circulating lipids, mimicked *in vitro* by loading cells using ox-LDL, which is known to activate PPARγ [52]. In parallel with the increased lipid uptake, an increased efflux of those lipids is induced by CLA *via* upregulation of the LXRα target *ABCA-1*. This is in keeping with previous studies where it has been established that increased PPARγ activity results in an increase in *CD36* and a decrease in *SRA-1* expression [52] and that a crosstalk between the two nuclear receptors, PPARγ and LXRα, induces a *CD36*-mediated positive regulation of *ABCA-1* [53-55]. Interestingly, during early stages of macrophage differentiation, we show that the PPARγ agonist significantly reduced *ABCA-1* (data not shown), whilst, in foam cells, *ABCA-1* expression is rescued to the control level. This is likely due to the fact that ox-LDL activates the *PPARγ* nuclear receptor, therefore, its agonist, mediates the restoration to a basal *ABCA-1* expression as previously documented [56,57].

Under normal homeostatic conditions, lipid that is taken into the macrophage can be efficiently metabolised and trafficked out of the cell *via* the RCT system, resulting in a balance between lipid uptake and efflux. In an atherogenic environment, however, where there are high concentrations of ox-LDL, there is a continuous influx of lipid and the increased intracellular concentration of free cholesterol overwhelms the RCT system. Excess cholesterol becomes esterified into cholesterol esters, which form lipid droplets, the distinctive features of foam cells. Therefore, to further confirm the proposed mechanism through which CLA inhibits foam cell formation, intracellular cholesterol content was analysed. *c*-9,*t*-11-CLA and CLA blend, significantly decreased the levels of both free and esterified cholesterol, confirming their specificity in atheroprotection, and reduced ox-LDL uptake, preventing intracellular accumulation of esterified cholesterol, whilst simultaneously, inducing free cholesterol efflux. The net effect of CLA, which primes the macrophages towards an MΦ2 phenotype prior to foam cell challenge, is altering cholesterol trafficking and lipid storage. Based on the data presented, it is feasible to suggest that CLA induces the macrophage to adopt an an anti-inflammatory phenotype which limits foam cell formation. To further address the inflammatory profile of CLA primed macrophages, the effect of CLA on cytokine generation was investigated.

The role of cytokine microenvironment on cholesterol metabolism has been extensively studied, playing a fundamental role in the priming of MΦ1/MΦ2 macrophage subtypes [41]. Therefore, macrophage generation of pro-inflammatory cytokines, namely, IL-1β, IL-6, IL-12p70, INF-γ and TNF-α were quantified, following CLA treatment of HPBMC-derived macrophages. Overall, in both macrophage and foam cells, the generation of most of the pro-inflammatory cytokines was prevented by CLA *via* a PPARγ-dependent mechanism. In general, the CLA conferred a “resolving” macrophage phenotype [11]. Moreover, it has been shown that IL-10 prevents pro-inflammatory cytokine production by activated macrophages, as part of its “deactivation” programme [58]. Supporting this is the observation that IL-10 production has been shown to be predominantly found in CD14^low^/CD16^high^ or “anti-inflammatory” monocytes [59]. Indeed our recent work demonstrated that IL-10 signalling pathway was modified during CLA-induced regression in murine model [17]. In keeping with this, we show that the atheroprotective isomer *c*-9,*t*-11-CLA increases macrophage and foam cell IL-10 production. However, this is not a PPARγ-mediated effect, as TROG actually decreases IL-10 generation in HBPMC-derived macrophage and has no effect in foam cells. Similarly, it is also unlikely, that this anti-inflammatory effect is due to an LXRα activation, as it has been shown that the LXRα agonist, T1317, does not increase levels of IL-10 in CD4-positive T cells [60]. Further studies are needed to investigate the exact mechanism of action of the CLA atheroprotective effect on the anti-inflammatory cytokine generation, which will likely identify additional PPARγ/LXRα-independent pathways.

Among the pro-inflammatory cytokine panel analysed, INF-γ was the only one which was decreased in both macrophage and foam cells by the CLA blend, strongly suggesting a PPARγ-dependent mechanism regulating the inhibition of pro-inflammatory mediators. This hypothesis is supported by a study where the PPARγ antagonist GW9662 reversed the decreased expression of pro-inflammatory cytokines TNFα, IL-1β and IL-6 and increased levels of the immunosuppressive cytokine TGF-β in M2-polarized THP-1 macrophages [61]. It has been extensively shown that INF-γ increases the expression of the scavenger receptor SRA-1, in both THP-1- and HBPMC-derived macrophages [62], and that DNA binding activity is most likely responsible for the IFN-γ-dependent expression of SRA-1. In addition, INF-γ decreases *ABCA-1* expression in murine peritoneal macrophages [63], decreasing cholesterol efflux, through pathways that include the upregulation of ACAT and the downregulation of efflux proteins. Based on these findings, IFN-γ can shift the equilibrium between macrophages and foam cells and, thus, impact the progression of an atherosclerotic lesion [64]. Moreover, it has been shown that LXRα activation also inhibits pro-inflammatory cytokine mRNA expression. In human lymphocytes the LXRα agonist, T1317 decreased INF-γ, TNF-α and IL-2 levels [60]. This is in keeping with our data, which shows that CLA mediates its effect *via* both a PPARγ and LXRα dependent mechanisms and decreases macrophage and foam cell generation of IFN-γ.

## Conclusions

**Figure 6:**
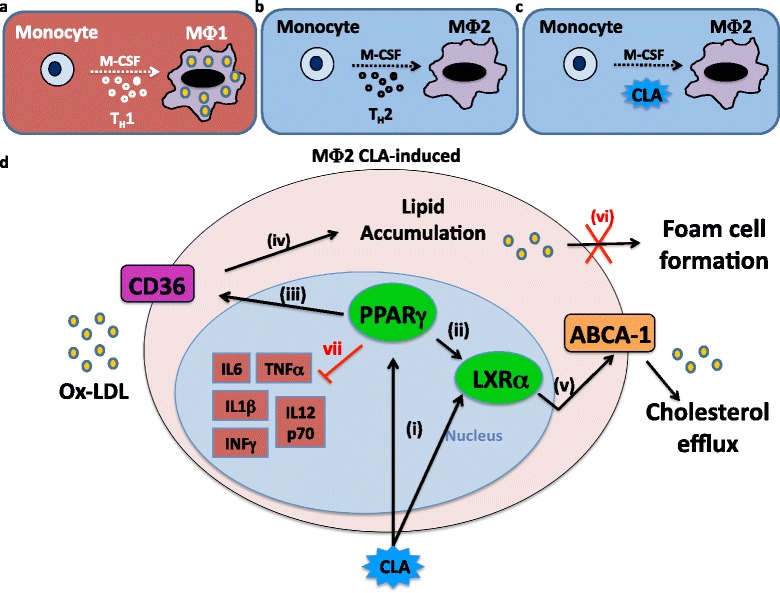
**Potential atheroprotective mechanism of CLA on the macrophage/foam cell axis. (a)** Th-1 cytokine environment primes M-CSF-triggered monocyte differentiation towards an MΦ1 macrophage pro-inflammatory phenotype. **(b)** Th-2 cytokine environment primes M-CSF-triggered monocyte differentiation towards an MΦ2 macrophage anti-inflammatory phenotype. **(c)** CLA action primes M-CSF-triggered monocyte differentiation towards an MΦ2-type macrophage. **(d)** In the presence of high levels of lipids in the extracellular matrix, CLA induces a dual mechanism PPARγ/LXRα-mediated (i-ii), by increasing *CD36* levels (iii), allowing lipids to enter the cell (iv), and secondly, promoting cholesterol efflux, by increasing *ABCA-1* mRNA expression (v), thus preventing lipid engulfment of the cell (vi), and the consequent foam cell formation. Moreover, CLA inhibits the secretion of pro-inflammatory cytokines (vii).

In summary, the data provides a novel mechanism for CLA in atherosclerosis Figure 6. The atheroprotective *c*-9,*t*-11 isomer and the CLA blend negatively regulate expression of pro-inflammatory mediators *via* a PPARγ and LXRα dependent mechanism. As a consequence, differentiation process is shifted towards an anti-inflammatory MΦ2 phenotype, characterised by increased *CD36* and *ABCA-1* expression, thus preventing macrophage lipid engulfment and promoting cholesterol efflux towards exogenous acceptors.

In conclusion CLA, by altering macrophage function, changes the relative distribution of macrophagic subsets, acquiring an anti-inflammatory phenotype which, ultimately, may impact on the lesion microenvironment, thus contributing to the mechanism through which CLA induces regression of pre-established atherosclerosis.

## Additional files

Additional file 1:
***In vitro*** **model of HBPMCs-derived macrophage differentiation.**
Additional file 2:
**Human Syber Green primer sequences.**
Additional file 3:
**Specificity of signals for co-incubation of CD68 and MR in HPBMC-derived macrophages.**
Additional file 4:
**CLA reduces foam cell formation in primary human cells.**
Additional file 5:
**LXR-a agonists increase LXRa and target gene ABCA-1 expression.**

## Abbreviations

ABCA1: ATP binding cassette A1
ACAT: Acetyl-coenzyme A cholesterol acetyl-transferase
CLA: Conjugated linoleic acid
FC: Free cholesterol
GSK: GSK2033
GW: GW9662
HPBMC: Human peripheral blood mononuclear cell
HS: Human Serum
IL: Interleukin
LA: Linoleic acid
LXRα: Liver X receptor-alpha
M-CSF: Macrophage colony stimulating factor
MΦ1/2: Macrophage phenotype 1/2
MR: Mannose receptor
OA: Oleic acid
ox-LDL: Oxidized low density lipoprotein
PMA: Phorbol 12-myristate 13-acetate
PPAR: Peroxisome proliferator activated receptor
RCT: Reverse cholesterol transport
SFM: Serum free media
SR: Scavenger receptor
T1317: T0901317
TC: Total cholesterol
Th1/2: T helper Lymphocyte 1/2
TNF-α: Tumor necrosis factor-α
TROG: Troglitazone
25-OH: 25-Hydroxy-cholesterol
