# Supplementary material for: Conjugated linoleic acid induces an atheroprotective macrophage MΦ2 phenotype and limits foam cell formation
Source: J Inflamm (Lond). 2015 Feb 19;12:15. doi: 10.1186/s12950-015-0060-9 (PMC4340802; doi:10.1186/s12950-015-0060-9)
Supplement: Additional file 1: — In vitro model of HBPMCs-derived macrophage differentiation. [file 12950_2015_60_MOESM1_ESM.pdf]

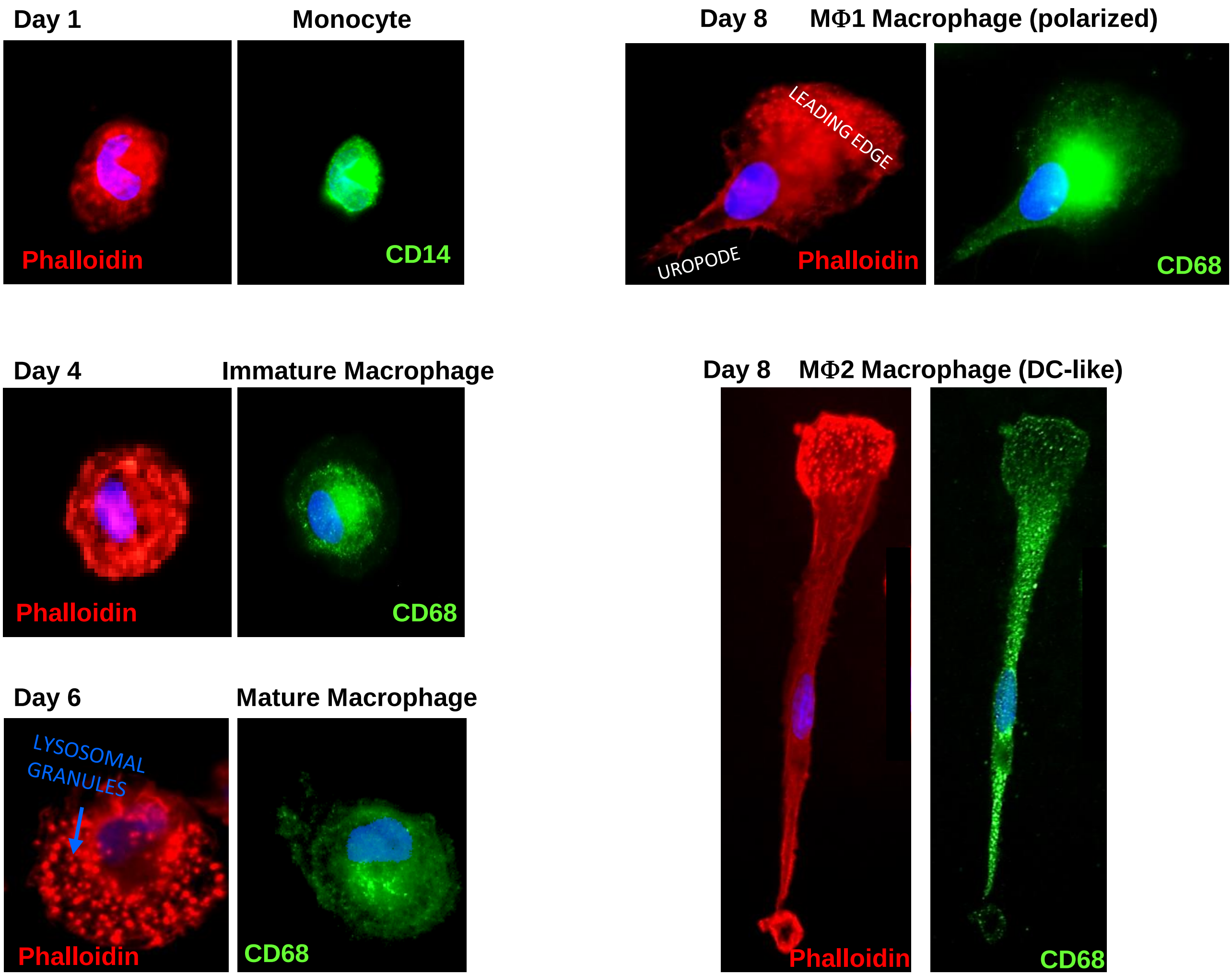


**Additional file 1 - *In vitro* model of HBPMCs-derived macrophage differentiation.** Differentiation of freshly isolated HBPMCs was stimulated using 100 ng/ml M-CSF. On the day of isolation (day 1) the monocyte phenotype is a classical phenotype with rounded cytoplasm and kidney shaped nuclei. After four days of culture, immature macrophages appeared with a more irregular shape and increased CD68 expression. At day 6, lysosomal granules are evident throughout the cytoplasm, and cytoskeletal rearrangements starts occur. At day 8, differentiation process is complete and lysosomal granules move to the leading edge of the macrophage, whereas, on the opposite site of the cell, fully formed pseusopodes appear. At this stage, macrophage heterogeneity is visible with differing phenotypes. For epifluorescent microscopy, nuclei were stained with DAPI (blue), F-actin with Alexa Fluor 647-Phalloidin (red). Anti-CD14 antibody was used as monocyte marker (visualized by secondary AlexaFluor 488) (green) and an anti-CD68 was used as macrophage marker (visualised by secondary Alexa Fluor 568) (green). Epifluorescent microscope images (63x magnification) are representative of three independent experiments
