# Supplementary material for: Conjugated linoleic acid induces an atheroprotective macrophage MΦ2 phenotype and limits foam cell formation
Source: J Inflamm (Lond). 2015 Feb 19;12:15. doi: 10.1186/s12950-015-0060-9 (PMC4340802; doi:10.1186/s12950-015-0060-9)
Supplement: Additional file 2: — Human Syber Green primer sequences. [file 12950_2015_60_MOESM2_ESM.pdf]

Human SYBR Green Primers	
**Gene**	**Sequence**
**ABCA1**	**Sense GCAGCAGAGCGAGTACTTCGTT**
	**Antisense CAAGACTATGCAGCAATGTTTTTGT**
**CD36**	**Sense TGTAACCCAGGACGCTGAGG**
	**Antisense GAAGGTTCGAAGATGGCACC**
**CD14**	**Sense CGCTCCGAGATGCATGTG**
	**Antisense AACGACAGATTGAGGGAGTTCAG**
**CD68**	**Sense GCTACATGGCGGTGGAGTACAA**
	**Antisense ATGATGAGAGGCAGCAAGATGG**
**CD163**	**Sense CGAGTTAACGCCAGTAAGG**
	**Antisense GAACATGTCACGCCAGC**

Additional file 2. Human Syber Green primer sequences. Relative gene expression quantification by RT-PCR was performed on an ABI Prism 7900HT Sequence Detection System (Applied Biosystems Inc., UK). MR and SRA-1 expression were examined using specific Taqman assays (Applied Biosystems Inc., UK), whilst, ABCA-1, CD36, CD14, CD68 and CD163 target genes were measured using specific Syber green assays (Applied Biosystems Inc., UK). Ct values were then normalised to 18s ribosomal RNA. In this table, sense (forward primer) and antisense (reverse primer) Syber Green primer sequences are displayed
