# Supplementary material for: Conjugated linoleic acid induces an atheroprotective macrophage MΦ2 phenotype and limits foam cell formation
Source: J Inflamm (Lond). 2015 Feb 19;12:15. doi: 10.1186/s12950-015-0060-9 (PMC4340802; doi:10.1186/s12950-015-0060-9)
Supplement: Additional file 3: — Specificity of signals for co-incubation of CD68 and MR in HPBMC-derived macrophages. [file 12950_2015_60_MOESM3_ESM.pdf]

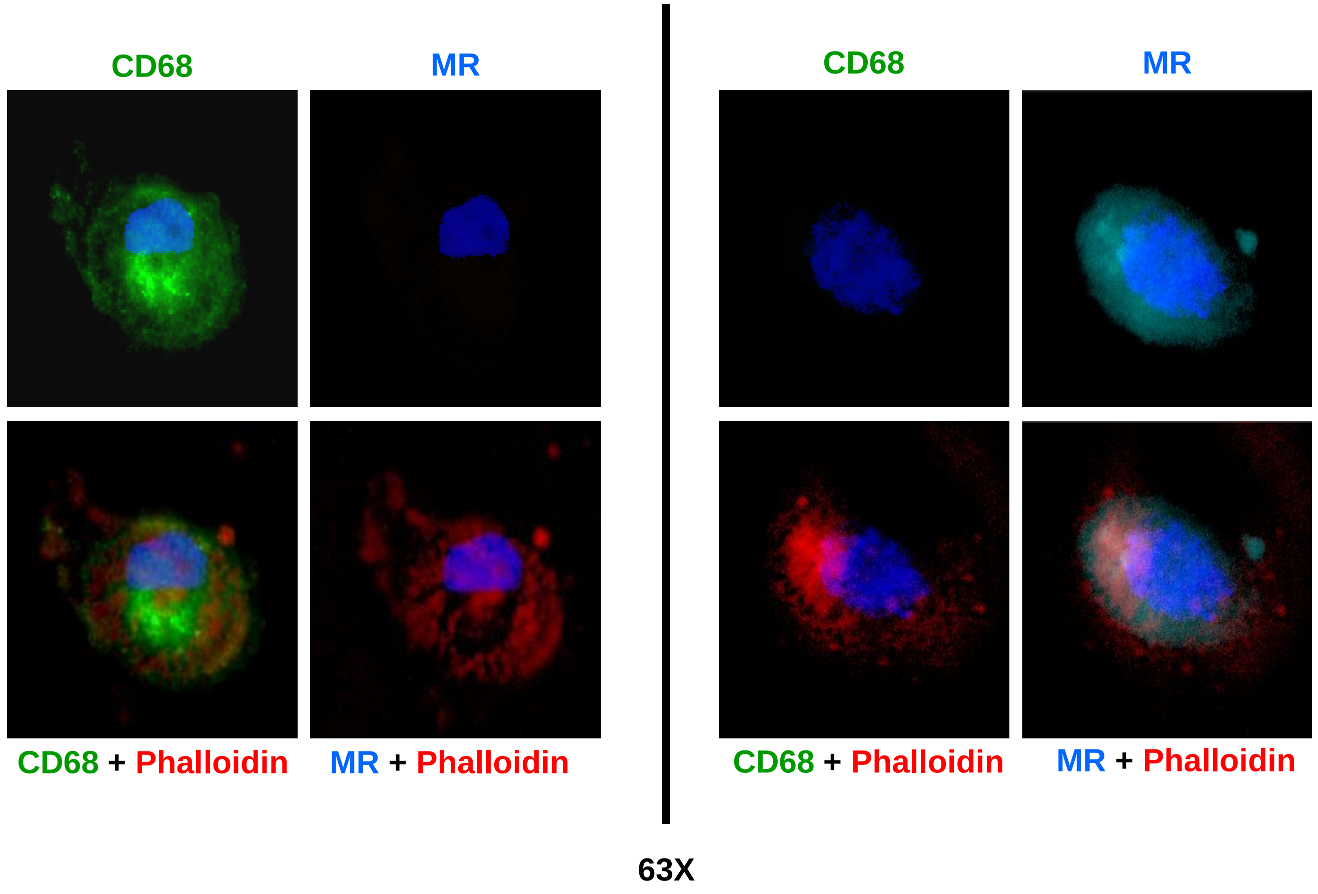

Additional file 3 - Specificity of signals for co-incubation of CD68 and MR in HPBMC-derived macrophages. a) Micrographs show positive staining at 488nm (top left panel) and no staining at 568nm with cells incubated with CD68 + AlexaFluor 488 antibodies (top left panel, green). **b)** Positive staining at 568nm (top right panel) and no staining at 488nm with cells incubated with MR + AlexaFluor 568 antibodies (top right panel, cyan). Bottom panels indicates co-incubation of CD68 or MR with F-actin stained with AlexaFluor 647-conjugated Phalloidin (red). Nuclei are DAPI stained (blue)
