# Supplementary material for: Conjugated linoleic acid induces an atheroprotective macrophage MΦ2 phenotype and limits foam cell formation
Source: J Inflamm (Lond). 2015 Feb 19;12:15. doi: 10.1186/s12950-015-0060-9 (PMC4340802; doi:10.1186/s12950-015-0060-9)
Supplement: Additional file 4: — CLA reduces foam cell formation in primary human cells. [file 12950_2015_60_MOESM4_ESM.pdf]

a

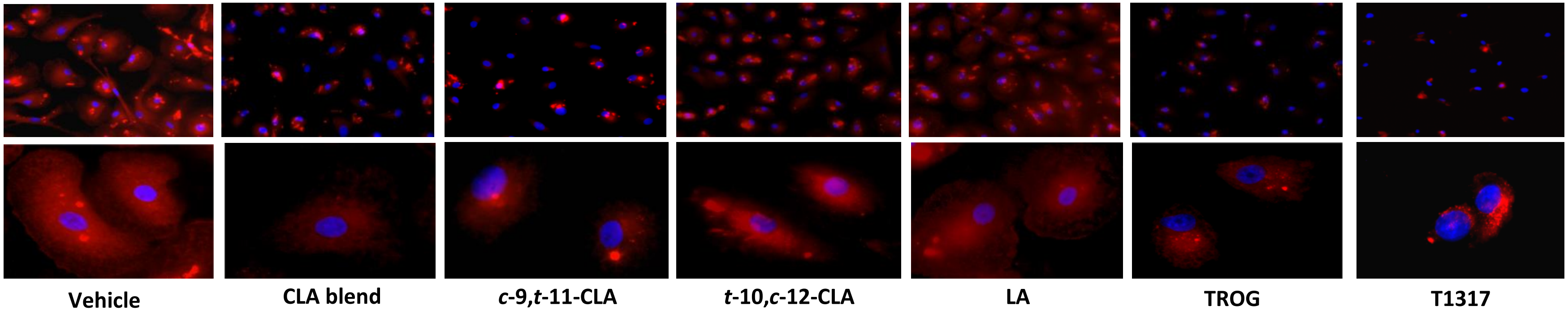

b

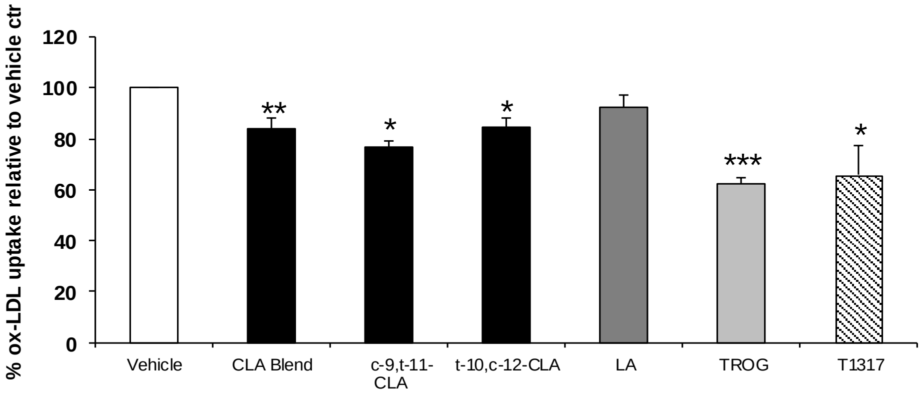

Additional file 4. CLA reduces foam cell formation in primary human cells. HPBMC-derived MCSF-induced macrophages were treated for 48 hrs with CLA or controls and then loaded with 50 ug/ml Dil-ox-LDL for 4 hrs to induce foam cell formation. Both CLA isomers and their blend inhibit ox-LDL accumulation, as showed by immunofluorescence **(a)** and quantified by spectrophotometric measurement **(b)** of ox-LDL fluorescence signal (adjusted per cell number), *via* a PPAR-γ (TROG) and LXR-α (T1317) dependent mechanism. 20x and 63x magnification of DAPI and Dil-ox-LDL stained foam cells are shown in **(a)**. Micrographs are representative of three independent experiments. Data are expressed as mean % uptake ± SEM where * = $p<0.05$, ** = $p<0.01$, *** = $p<0.001$ *vs* DMSO vehicle control
