# Supplementary material for: Conjugated linoleic acid induces an atheroprotective macrophage MΦ2 phenotype and limits foam cell formation
Source: J Inflamm (Lond). 2015 Feb 19;12:15. doi: 10.1186/s12950-015-0060-9 (PMC4340802; doi:10.1186/s12950-015-0060-9)
Supplement: Additional file 5: — LXR-a agonists increase LXRa and target gene ABCA-1 expression. [file 12950_2015_60_MOESM5_ESM.pdf]

a

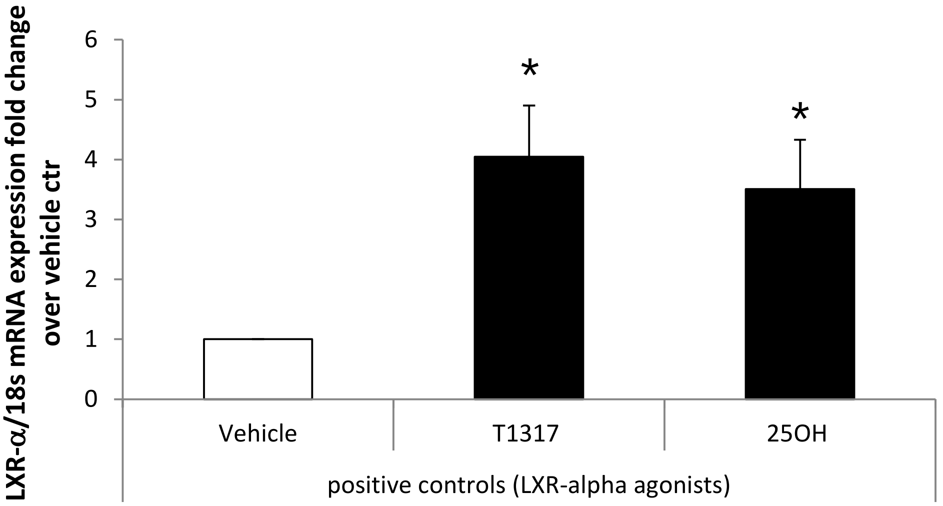

b

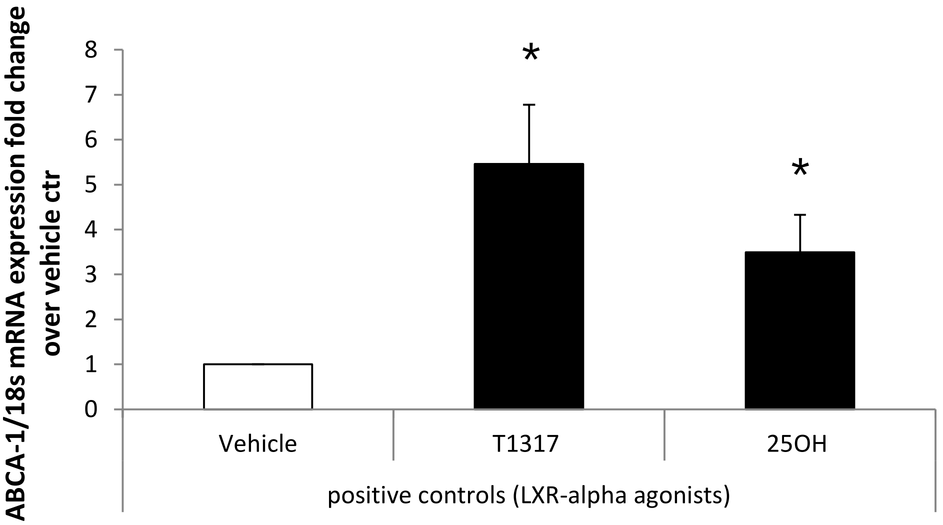

Additional file 5. LXR-α agonists increase LXRα and target gene ABCA-1 expression. RT-PCR analysis of **(a)** LXR-α and **(b)** ABCA-1 mRNA expression, following treatment of HPBMC-derived macrophages with LXR-α agonists (T1317 and 25-OH) used as positive controls in subsequent experiments. Statistical analysis of three independent experiments is expressed as fold change expression over control, where *$p<0.05$ *vs* DMSO vehicle control
